# The Correlation between Peripartum Cardiomyopathy and Autoantibodies against Cardiovascular Receptors

**DOI:** 10.1371/journal.pone.0086770

**Published:** 2014-01-23

**Authors:** Jiamei Liu, Yidan Wang, Mulei Chen, Wenshu Zhao, Xin Wang, Hua Wang, Zhiyong Zhang, Juan Zhang, Lin Xu, Jin Chen, Xinchun Yang, Lin Zhang

**Affiliations:** Heart Failure Center, Department of Cardiology, Beijing Chao-Yang Hospital, Capital Medical University, Beijing, China; University of Torino, Italy

## Abstract

**Background:**

Peripartum cardiomyopathy (PPCM) is characterized by left ventricular systolic dysfunction and heart failure. However, its pathogenesis is not clear. Our preliminary study revealed that autoantibodies against β_1_-adrenergic receptors (β_1_R-AABs) and M_2_-muscarinic receptors (M_2_R-AABs) participated in heart failure regardless of primary heart disease. Whether β_1_R-AABs and M_2_R-AABs participate in the pathogenesis of PPCM is still unknown.

**Methods:**

Totally 37 diagnosed PPCM patients and 36 normal pregnant women were enrolled in this study. Clinical assessment and 2-dimensional echocardiographic studies as well as the measurement of β_1_R-AABs or M_2_R-AABs by enzyme linked immunosorbent assay (ELISA) were performed.

**Results:**

The positive rates for β_1_R-AABs and M_2_R-AABs were 59.5% (22/37) and 45.9% (17/37) in PPCM patients, and 19.4% (7/36) (*P*<0.001) and 16.67% (6/36) (*P*<0.001) in normal pregnant women, respectively. Both β_1_R-AABs and M_2_R-AABs had a positive correlation with serum expression level of NT-proBNP, left ventricular dimension and NYHA FC (rs: 0.496–0.892, *P*<0.01). In addition, a negative correlation between the activity of β_1_R-AABs and M_2_R-AABs and LVEF, LVFS was observed (rs: −0.488–0.568, *P*<0.01). Moreover, autoantibodies against cardiovascular receptors increased the risk of the onset of PPCM (OR = 18.786, 95% confidence interval 1.926–183.262, *P* = 0.012).

**Conclusions:**

The β_1_R-AABs and M_2_R-AABs reveal a significant elevation and are correlated with the increased left ventricular dimension and worse cardiac contraction function. The autoantibodies of cardiovascular receptors are independent risk factors for the onset of PPCM.

## Introduction

Peripartum cardiomyopathy (PPCM) is a serious disease with unknown etiology, which occurs between the last month of pregnancy and 5-month puerperium with significant morbidity and mortality. The diagnostic criteria include: (1) The disease occurs between the last month of pregnancy and 5-month puerperium; (2) There has no definite reason for heart failure; (3) LVEF <45%, LVFS <30%. The reported incidence fluctuates among geographic regions, but it is higher in Africa. It has been estimated to be 1∶3000–4000 in USA, 1∶400 in Haiti, and 1∶1000 in South Africa [1]. Although the mechanism of PPCM is still unknown, increasing evidences suggest that autoimmunity may play an important role in the development of PPCM [2].

The role of autoimmunity in cardiovascular diseases has become one of the focal points in the field of cardiomyopathy and heart failure. The β_1_-adrenergic and M_2_-muscarinic receptors belong to the family of cardiac G-protein-coupled receptors. Circulating autoantibodies against the second extracellular loop of β_1_-adrenergic receptors and M_2_-muscarinic receptors have been detected in a number of cardiovascular diseases characterized by heart failure including idiopathic dilated cardiomyopathy (IDCM) [3] and chronic Chagas’ heart disease (ChHD) [4]. PPCM patients have been confirmed to be positive for β_1_R-AABs [5]. Our previous studies have also demonstrated that β_1_R-AABs and M_2_R-AABs are highly prevalent and participated in the pathogenesis of dilated cardiomyopathy. In addition, the density of β_1_R-AABs and M_2_R-AABs is significantly higher in patients with heart failure regardless of the primary heart disease [6]. The purpose of this study is to determine whether PPCM is correlated with the serum levels of β_1_R-AABs and M_2_R-AABs.

## Results

### Study Characteristics

The characteristics of the study population at the first presence in cardiac clinic (baseline) are shown in Table 1. All these patients were diagnosed as PPCM for the first time. Totally 13 of 37 patients were primiparous, and 21 of 37 patients with symptoms in the postpartum period: all within three months after delivery. At baseline, one patient was in NYHA FC II, 16 patients in FC III and 20 patients in FC IV. The clinical profiles of the study population including patients with PPCM and normal pregnant group were summarized in Table 2. Compared with normal pregnant women, PPCM patients had a higher heart rate and blood pressure (*P*<0.05). Left ventricular dimension was significantly enlarged and left ventricular ejection fraction coincided with left ventricular fractional shortening was significantly decreased (*P*<0.001).

**Table 1:** Baseline characteristics of PPCM patients.

Parameters	Value
**Age (years)**	29±6
**Parity (range)**	1 (1–3)
**Preeclampsia**	18 (48.6)
**NYHA functional class (n, %)**	
II	1 (2.7)
III	16 (43.2)
IV	20 (54.1)
**Beginning of symptoms**	
Ante partum	16
1–3 month post-partum	21
**Blood pressure (mmHg)**	
Systolic	131±13
Diastolic	86±8
**Heart rate (beats/min)**	100±19
**Echocardiographic data**	
Left-ventricular EDD (mm)	59±6
Left-ventriculer ESD (mm)	47±7
Ejection fraction (%)	38±7
Fractional shortening (%)	19±4

Data were expressed as numbers (%), mean ± SD, or medians (interquartile range, IQR),

EDD = end-diastolic diameter, ESD = end-systolic diameter, NYHA = New York Heart Association.

**Table 2:** Clinical profiles of study population.

Parameters	PPCM (n = 37)	NP (n = 36)	*P* value
**Age (years)**	29±6	28±3	0.867
**Blood pressure (mmHg)**			
Systolic	131±13	115±7	**<0.001**
Diastolic	86±8	73±6	**<0.001**
**Heart rate (beats/min)**	100±19	89±12	**0.027**
**NT-proBNP (pg/mL)**	2901.00(1116.00,7078.00)	113.42(95.24,162.00)	**<0.001**
**Echocardiographic data**			
Left-ventricular EDD (mm)	59±6	45±5	**<0.001**
Left-ventriculer ESD (mm)	47±7	27±4	**<0.001**
Ejection fraction (%)	38±7	69±5	**<0.001**
Fractional shortening (%)	19±4	41±4	**<0.001**

Data were expressed as numbers (%), mean ± SD, or medians (interquartile range, IQR),

PPCM = peripartum cardiomyopathy, NP = normal pregnant, NT-proBNP* = *N-terminal pro-brain natriuretic peptide, EDD = end-diastolic diameter, ESD = end-systolic diameter.

### Autoantibody Screening

The positive sera for β_1_R-AABs were observed in 59.5% (22/37) of PPCM patients, while 19.4% (7/36) (*P*<0.001) in normal pregnant women. The positive sera for M_2_R-AABs were 45.9% (17/37) in PPCM patients, while 16.67% (6/36) (*P*<0.001) in normal pregnant women. Moreover, in positive cases, autoantibody titer (geometric mean) was higher in patients with PPCM when compared with normal pregnant women both for β_1_R-AABs and M_2_R-AABs. The geometric mean titers for β_1_R-AABs and M_2_R-AABs were 1∶129 and 1∶143, while they were 1∶80 (*P* = 0.003) and 1∶53 (*P*<0.001) in normal pregnant women. Totally 17 of 37 (46%) PPCM patients were positive for both β_1_R-AABs and M_2_R-AABs. Among other 19 patients, 15 (40%) patients were negative for β_1_R-AABs and M_2_R-AABs, and 5 (14%) patients were positive for β_1_R-AABs.

### Follow-up Examination

One patient (3%) died during the study period. The death was due to the progression of heart failure. Since the death was due to the progression of heart failure, patients who died were classified in FC IV at the latest follow-up examination. All patients received standard therapy for heart failure, which included daily 0.125 mg of digitalis, furosemide [median daily dose 40 mg (20–40 mg)], perindopril [median daily dose 4 mg (2–4 mg)], and betaloc [median daily dose 25 mg (6.25–50 mg)].

### LV Function, Dimension and Heart Rate

Patients who completed 12 months of standard therapy showed a significant reduction in heart rate, LV dimension, and significant improvement in echocardiographic LVEF and LVFS (*P*≤0.001) and NYHA FC (*P*<0.001). Normalization of LVEF (≥50%) was observed in 31 (86.1%) patients. Clinical data and NYHA FC were compared at the first presence in the cardiac clinic at baseline and after 12 months of standard therapy as described in Table 3.

**Table 3:** Clinical variables, left-ventricular dimension and functional class from 36 patients who completed the 12-month trial.

	Baseline	12 months	*P* value
**NYHA functional class**			
I	0	18	**<0.001**
II	1	17	**<0.001**
III	16	1	**<0.001**
IV	20	0	**<0.001**
**Blood pressure (mmHg)**			
Systolic	131±13	115±11	**<0.001**
Diastolic	86±8	75±6	**<0.001**
**Heart rate (beats/min)**	100±19	72±8	**<0.001**
**NT-proBNP(pg/mL)**	2901 (1116, 7078)	432 (34,1268)	**<0.001**
**Echocardiographic data**			
Left-ventricular EDD (mm)	59±6	49±5	**<0.001**
Left-ventricular ESD (mm)	47±7	34±6	**<0.001**
Ejection fraction (%)	38±7	62±8	**<0.001**
Fractional shortening (%)	19±4	31±4	**<0.001**

Data were expressed as numbers (%), mean ± SD, or medians (interquartile range, IQR),

NYHA = New York Heart Association, EDD = end-diastolic diameter, ESD = end-systolic diameter, NT-proBNP* = *N-terminal pro-brain natriuretic peptide.

### Dynamic Variation of Autoantibody

**Figure 1:**
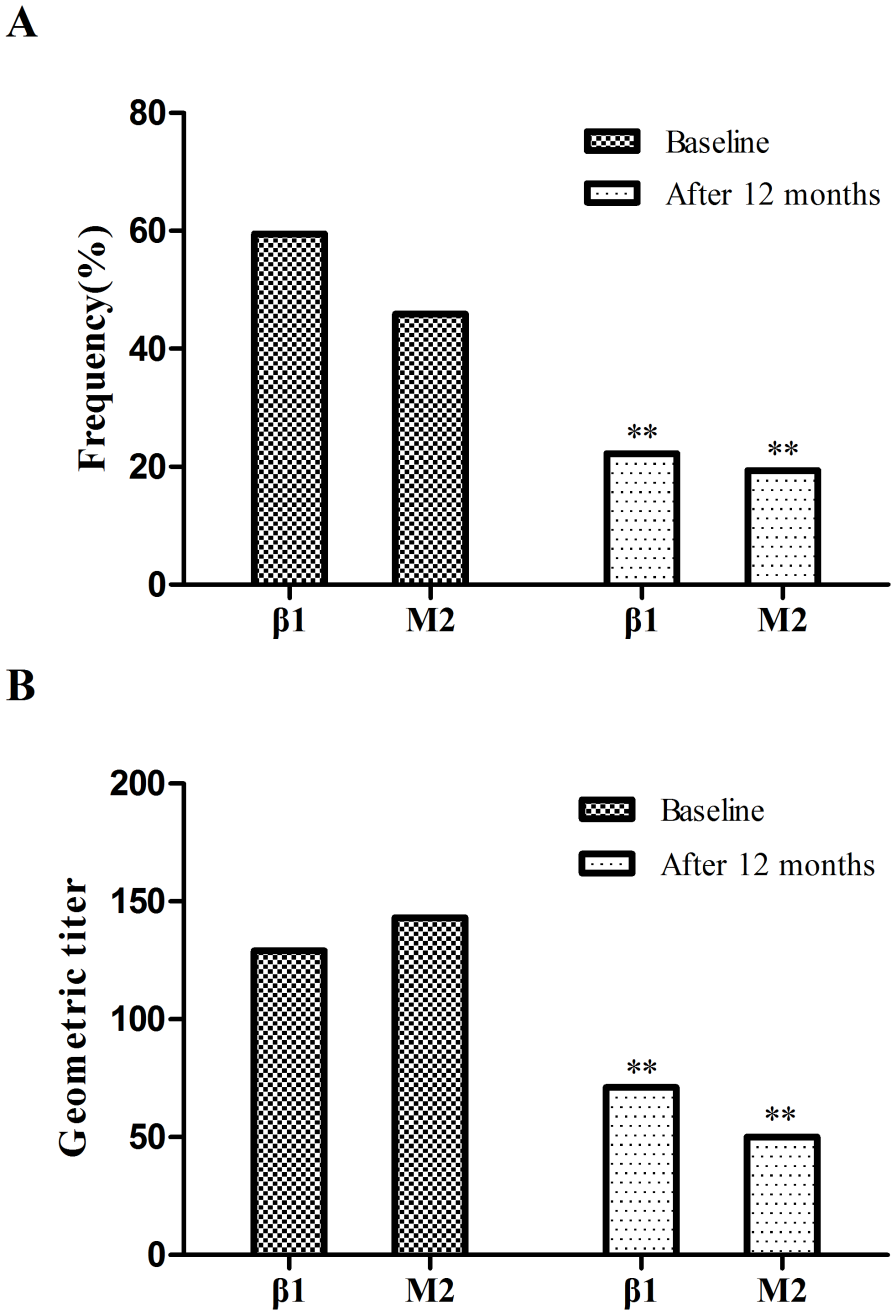
The comparison for frequency and geometric titers of autoantibody between baseline and after 12-month treatment. After 12-months treatment, the frequency and geometric titers of both β_1_R-AABs and M_2_R-AABs were decreased significantly (*P*<0.001).

After 12 months treatment, the positive rate for β_1_R-AABs declined from 59.5% to 22.2% (*P*<0.001), as well as M_2_R-AABs declined from 45.9% to 19.4% (*P*<0.001). The geometric means of autoantibodies were also decreased significantly (*P*<0.001). The geometric mean titers for β_1_R-AABs and M_2_R-AABs were declined from 1∶129 to 1∶71 and 1∶143 to 1∶50, respectively (Figure 1).

### Comparison of Echocardiographic Data from Patients with Different Positive Autoantibodies

**Figure 2:**
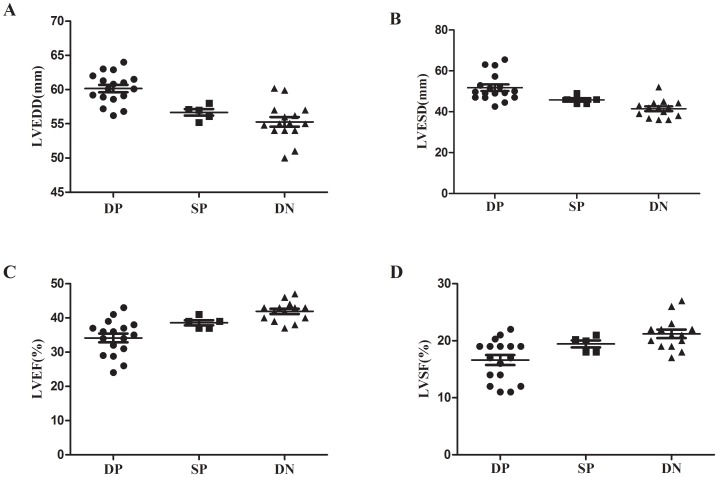
The comparison of echocardiographic data from patients with different positive autoantibodies. Echocardiographic data among patients with double positive (DP) autoantibodies, single positive autoantibodies (SP) and double negative (DN) autoantibodies had significant difference: LVEDD and LVESD: DP>SP>DN (*P = *0.001), LVEF and LVSF: DP<SP<DN (*P*<0.01).

There were 17 (46%) patients with double positive (DP) autoantibodies, 5 (14%) patients with single positive (SP) autoantibodies and 15 (40%) patients with double negative (DN) autoantibodies. Echocardiographic data among three groups had significant difference: left ventricular end diastolic dimension (LVEDD): 62.5±7.1 vs. 56.9±1.0 vs. 54.8±4.3, (χ^2^ = 14.411, *P* = 0.001); left ventricular end systolic dimension (LVESD): 51.2±7.5 vs. 45.8±2.1 vs. 42.2±4.3, (χ^2^ = 14.944, *P = *0.001); left ventricular ejection fraction (LVEF): 33.7±7.9 vs. 38.6±1.7 vs. 41.9±2.9, (χ^2^ = 11.879, *P = *0.003); left ventricular fractional shortening (LVFS): 16.8±4.0 vs. 19.4±1.4 vs. 21.2±2.8, (χ^2^ = 9.717, *P* = 0.008). After standard treatment for 1 year, three groups had obvious improvement on echocardiography, however, the degree of improvement was different: LVEDD: 52.3±4.4 vs. 46.4±1.1 vs. 44.5±1.2, (χ^2^ = 18.837, *P*<0.001); LVESD: 38.4±5.1 vs. 32.4±1.1 vs. 28.4±2.9, (χ^2^ = 17.433, *P*<0.001); LVEF: 57.2±7.0 vs. 61.6±2.9 vs. 68.5±5.0, (χ^2^ = 13.715, *P = *0.001); LVFS: 28.5±3.4 vs. 30.4±2.1 vs. 33.9±2.4, (χ^2^ = 12.923, *P = *0.002) (Figure 2).

### Determinant of Serum Antibody Levels

The levels of serum β_1_R-AABs and M_2_R-AABs (both frequency and titer of autoantibody) were positively correlated with left ventricular dimension, NT-proBNP and NYHA FC (Spearman coefficient differs from 0.378 to 0.590, all *P*<0.05). Besides the levels of serum β_1_R-AABs and M_2_R-AABs (both frequency and titer of autoantibody) were negatively correlated with LVEF and LVSF (Spearman coefficient differs from −0.390 to −0.570, all *P*<0.05). A significant correlation was observed between β_1_R-AABs and M_2_R-AABs (rs = 0.886, *P*<0.001). The results were described in Tables 4 and 5.

**Table 4:** Correlation between serum anti-β_1_R-AAB levels and other parameters.

	Frequency of autoantibody		Titer of autoantibody	
	Spearman coefficient	*P* value	Spearman coefficient	*P* value
**NYHA FC**	0.892	**<0.001**	0.702	**<0.001**
**NT-proBNP(pg/mL)**	0.567	**<0.001**	0.581	**<0.001**
**Echocardiographic data**				
Left-ventricular EDD (mm)	0.578	**<0.001**	0.525	**0.001**
Left-ventricular ESD (mm)	0.601	**<0.001**	0.496	**0.002**
Ejection fraction (%)	−0.561	**<0.001**	−0.568	**<0.001**
Fractional shortening (%)	−0.488	**<0.001**	−0.499	**0.002**

NYHA FC = New York Heart Association functional class, NT-proBNP* = *N-terminal pro-brain natriuretic peptide EDD = end-diastolic diameter, ESD = end-systolic diameter.

**Table 5:** Correlation between serum anti-M_2_R-AAB levels and other parameters.

	Frequency of autoantibody		Titer of autoantibody	
	Spearman coefficient	*P* value	Spearman coefficient	*P* value
**NYHA FC**	0.736	**<0.001**	0.625	**<0.001**
**NT-proBNP (pg/mL)**	0.541	**<0.001**	0.554	**<0.001**
**Echocardiographic data**				
Left-ventricular EDD (mm)	0.607	**<0.001**	0.530	**0.001**
Left-ventricular ESD (mm)	0.622	**<0.001**	0.536	**0.001**
Ejection fraction (%)	−0.526	**0.002**	−0.532	**0.001**
Fractional shortening (%)	−0.498	**<0.001**	−0.528	**0.001**

NYHA FC = New York Heart Association functional class, NT-proBNP* = *N-terminal pro-brain natriuretic peptide EDD = end-diastolic diameter, ESD = end-systolic diameter.

### Correlation of β_1_R-AABs and M_2_R-AABs and the Onset of Peripartum Cardiomyopathy

Univariate analysis showed that several factors increased the risk for the onset of PPCM, including autoantibodies against cardiovascular receptors, advanced maternal age, multiple pregnancies, pregnancy-induced hypertension, and poor socioeconomic status. Multiple logistic analysis showed that the level of autoantibodies against cardiovascular receptors and pregnancy-induced hypertension were an independent predictor of the PPCM attack (autoantibodies against cardiovascular receptors: OR = 18.786, 95% confidence interval 1.926–183.262, *P = *0.012; pregnancy-induced hypertension: OR = 17.305, 95% confidence interval 1.061–282.299, *P* = 0.045). The result was shown in Table 6.

**Table 6:** Univariate and multivariate analysis for the onset of PPCM.

	Univariate analysis			Multivariate analysis		
	OR	95% CI	*P* value	OR	95% CI	*P* value
Autoantibody	28.6	5.979–138.186	**<0.001**	18.786	1.926–183.262	**0.012**
Advanced maternal age	2.635	1.007–6.898	**0.048**			
Multiple pregnancies	5.01	1.460–17.189	**0.010**			
Multiparity	3.259	0.351–35.517	0.284			
**Preeclampsia**	16.625	4.339–63.705	**<0.001**	17.305	1.061–282.299	**0.045**
Poor socioeconomic status	6.821	2.190–21.244	**0.001**			

## Discussion

### Major Findings

In this study, synthetic peptides corresponding to the sequences of the second extracellular loop of human β_1_ receptor (amino acid sequence number: 197–222) and M_2_ receptor (amino acid sequence number: 169–193) were used. We detected the autoantibodies against β_1_ and M_2_ receptors in patients’ sera with ELISA and demonstrated that the frequency and titer of serum β_1_R-AABs and M_2_R-AABs in patients with PPCM were higher than those of normal pregnant women. The comparison between β_1_R-AABs and M_2_R-AABs double positive group, single positive group and double negative group revealed that the expansion of left ventricular and decrease of heart function: double positive group> single positive group> double negative group. After treatment for 1 year, recovery of left ventricular dimension and heart function: double positive group< single positive group< double negative group. The test of β_1_R-AABs and M_2_R-AABs may have predicted value for the prognosis of PPCM. The levels (both frequency and titer) of serum β_1_R-AABs and M_2_R-AABs were positively associated with New York Heart Association functional class, left ventricular dimension and plasma NT-proBNP levels, and were negatively associated with left ventricular ejection fraction, left ventricular fractional shortening. Autoantibodies against cardiovascular receptors were independent predictors of the onset of PPCM. This study suggested that β_1_R-AABs and M_2_R-AABs play an important role in the pathogenesis of PPCM.

### β_1_R-AABs and M_2_R-AABs and Cardiovascular Disease

During the past few years, autoimmune mechanisms in heart disease have gained extensive attentions. Autoimmunity has been observed in several cardiovascular diseases such as dilated cardiomyopathy (DCM) and heart failure, rheumatic fever, myocarditis, atherosclerosis, and others [7]–[8]. Belonging to the family of G protein-coupled receptors, β_1_R and M_2_R are the major adrenergic and muscarinic receptors present in myocardial tissue, respectively [9]–[10]. β_1_R-AABs and M_2_R-AABs were first detected in the idiopathic dilated cardiomyopathy (IDCM) [11]–[12]. IDCM is characterized by systolic dysfunction and chamber dilatation of the left heart or both left and right ventricles as same as PPCM. Further studies have demonstrated that autoimmune mechanisms seem to play a major role in the pathogenesis of IDCM. Monthly immunization of rabbits for 1 year using synthetic peptides corresponding to the sequence of the second extracellular loop of either the human β_1_R or M_2_R could induce cardiac morphological changes including significantly enlarged ventricles and thinner walls, resembling the changes of IDCM in humans [13]. Others have attempted to conduct mouse immunization by using plasmid DNA encoding entire human β_1_AR or M_2_R proteins, which can lead to contractile dysfunction and cardiac remodeling [14].

Our preliminary study revealed that β_1_R-AABs and M_2_R-AABs not only existed in IDCM patients, but also in heart failure caused by different cardiac diseases. Moreover, the serum level of autoantibody has no significant difference among different heart diseases, which suggested that the generation of β_1_R-AABs and M_2_R-AABs had no relationship with primary heart disease, but was correlated with the damage of cardiac function.

### Autoimmune Mechanisms in PPCM

PPCM is characterized as systolic dysfunction and heart failure as well as IDCM. Its pathogenesis was still unclear. The autoimmune mechanism was believed to participate in the pathogenesis of PPCM. High titers of auto-antibodies against selected cardiac tissue proteins have been found in the majority of women with PPCM [15]. Warraich et al. investigated the role of humoral immunity and showed that class G and all subclass immunoglobulins against cardiac myosin heavy chain were raised in PPCM [16]. Recent studies further confirmed that β_1_R-AABs existed in the serum of PPCM patients, and was correlated with plasma NT-proBNP [5].

### Autoantibodies and PPCM

Previous studies revealed that β_1_R-AABs and M_2_R-AABs might lead to cardiac remodeling in cardiomyopathy. In the present study, significantly higher levels of β_1_R-AABs and M_2_R-AABs in the serum of PPCM than normal pregnant women were observed. For PPCM patients, the enlarged left ventricular chamber may return to normal level, which seems to improve better than IDCM. In our study, the titer of autoantibodies revealed a decline, which is coincided with the decrease of left ventricular and the improvement of heart function after treatments. Therefore, β_1_R-AABs and M_2_R-AABs may participate in the pathological change of cardiomyocytes. What’s more, these two autoantibodies were the independent risk factors for the attack of PPCM and may influence the prognosis of PPCM patients. All of these investigations suggested that β_1_R-AABs and M_2_R-AABs might participate in the pathogenesis of PPCM.

It’s interesting to notice that, a hypothesis currently being widely entertained is that PPCM may be a vascular disease [17]. Antiangiogenic factor (sFlt1), which is markedly elevated in preeclampsia, is involved in the pathogenesis of PPCM. Another novel pilot study has demonstrated for the first time that the presence of autoantibodies against β_1_ is increased in patients with preeclampsia [18], which is the risk factor for PPCM. Whether the autoantibodies against cardiovascular receptors are associated with the elevation of antiangiogenic factors, thus leading to that further studies of PPCM still are needed.

### Study Limitations

PPCM is a diagnosis by exclusion. As the objects of this study are pregnant women, the coronary angiography, and right and left catheterization are not allowed for application to exclude other kinds of cardiac diseases. Because the morbidity of PPCM is not very high in China, especially in Beijing, the case number is insufficient. As all these patients enrolled in our study were Chinese Han and body mass index (BMI) was not mentioned in this article, the assessment of autoantibodies as an independent risk factor for PPCM were incomplete, which could result in the continuous improvement in the future. What’s more, the predictive value of autoantibodies against cardiovascular receptors for the prognosis of PPCM needs further studies.

### Conclusion

This study has demonstrated that the presence of β_1_R-AABs and M_2_R-AABs were prevalent in a cohort of PPCM patients. The positive rates and the serum titers of β_1_R-AABs and M_2_R-AABs were positively correlated with left ventricular dimension, and negatively correlated with the cardiac contraction function of PPCM patients. The autoantibodies of cardiovascular receptors were independent risk factors for the onset of PPCM.

We propose that autoantibodies against cardiovascular receptors may be involved in the pathogenesis of PPCM. Further studies are needed to confirm these findings and dissect the underlying mechanisms for this novel observation.

## Materials and Methods

### Study Design and Patient Enrollment

The protocol as well as the consent form was approved by the Ethics Committee of Capital Medical University Beijing Chao-Yang Hospital and the study protocol complied with the Declaration of Helsinki. All participants provided written informed consent prior to admission into the study.

Since 1998, 37 Chinese Hanpatients with newly diagnosed PPCM were enrolled in Beijing Chao-Yang Hospital. The enrolled patients should meet following inclusion criteria: (1) age ≥16 or ≤40 years old, (2) New York Heart Association functional classes (NYHA FCs) II–IV, (3) symptoms of congestive heart failure developed in the last month of pregnancy or during the first 5 months post-partum, (4) no other identifiable causes for heart failure, (5) left ventricular ejection fraction (LVEF) <45%, left ventricular fractional shortening <30% by transthoracic echocardiography, (6) sinus rhythm and (7) eligible patients with high quality echocardiographic images. Exclusion criteria: (1) significant organic valvular heart disease, (2) systolic blood pressure (SBP) >160 mmHg and/or diastolic blood pressure >100 mmHg, (3) clinical conditions other than cardiomyopathy with increased autoantibody by screening the serum for rheumatoid arthritis and HIV and the evidence of sepsis, (4) severe anaemia (haemoglobin concentration <9 g/dL), (5) metabolic disorders affecting lipoprotein metabolism such as thyroid disease and (6) Clinical data are not complete. Clinical assessment, echocardiography and blood analysis were conducted at baseline and after 12 months of standard therapy. All patients received standard therapy for PPCM (β-blocker, angiotensin-converting enzyme inhibitors, diuretics and if indicated digoxin). Patients with an ejection fraction ≤25% or LV thrombus additionally received anticoagulation therapy. The follow-up examination at Beijing Chao-Yang Hospital was provided for all patients.

### Autoantibody Detection

The autoantibodies against β_1_-adrenergic and M_2_-muscarinic receptors were measured in patient sera through an enzyme-linked immunosorbent assay (ELISA) using a synthetic peptide corresponding to the sequence of the second extracellular loop of human β_1_ and M_2_ receptor (amino acid sequence number, β_1_∶197–222: H-W-W-R-A-E-S-D-E-A-R-R-C-Y-N-D-P-K-C-C-D-F-V-T-N-R; M_2_∶169–193: V-R-T-V-E-D-G-E-C-Y-I-Q-F-F-S-N-A-A-V-T-F-G-T-A-I). The peptide was synthesized by Biological Institute of the Chinese Academy of Medical Sciences (CAMS) & Peking Union Medical College (PUMC) using a solid-phase method of Merrifield, and the purity was evaluated by high-pressure liquid chromatography (HPLC) on an automatic amino acid analyzer (Beckman instrument, Inc, USA). The procedures of ELISA were as follows: 50 µL of peptide (5 mg/L) in 100 mmol/L Na_2_CO_3_ solution (pH = 11.0) was incubated overnight at 4°C on NUNC (Kastrup, Denmark) microtiter plates. The wells were saturated with phosphate-buffered saline (PBS: 10 mmol/L phosphate, 140 mmol/L NaCl, pH 7.3–7.4) supplemented with 0.01% (w/v) Thimerosal (Sigma, St. Louis MO, USA), 0.1% (v/v) Tween-20 (Sigma, St. Louis, MO, USA) (PBS-T) and 10% (v/v) fetal bovine serum (PMT). A total of 50 µL of patient serum with gradient dilutions from 1∶20 to 1∶160 was added to saturated microtiter plates and incubated for 1 h at 37°C. After the wells were washed with PBS-T for 3 times, an affinity-purified biotinylated rabbit anti-human IgG (H+L) antibody (Jackson Laboratories, PA, USA) (1∶500 dilution in PMT) was added and allowed to react for 1 h. After washing the wells for 3 times with PBS-T, the bound biotinylated antibody was detected by incubating the plates for 1 h with streptavidin-peroxidase (2 ml/L) (Jackson Laboratories, PA, USA) solution in PMT, which was followed by washing for 3 times with PBS and the addition of substrate for peroxidase 2.5 mmol/L H_2_O_2_-2 mmol/L ABTS (Sigma, St. Louis, MO, USA). Optical density (OD) was recorded after 30 min at 405 nm in a micro plate reader (Molecular Devices Corp, Menlo Park, CA, USA). Positive rate was defined as positive/negative [P/N = (sample OD-blank OD)/blank OD)] ≥2.1 [19]–[21]. The titer of autoantibody is the highest degree of the sample with P/N≥2.1 when diluted from 1∶20 to 1∶160.

### Echocardiographic Studies

All studies were performed and interpreted by the same operator who was blinded to the protocol. Two-dimensional targeted M-mode echocardiography with Doppler color flow mapping was performed for every patient. Left ventricular dimension was measured according to the American Society of Echocardiography guidelines [22]. Measurements of LV dimension and function were determined on an average of ≥3 beats. Diastolic mitral flow was assessed by pulsed-wave Doppler echocardiography from the apical four-chamber view. E-wave deceleration time was measured as the interval between the early peak diastolic velocity and the point at which the steepest deceleration slope was extrapolated to the zero line.

### Statistical Methods

The measured data were expressed as mean ± SD or median (interquartile range, IQR) and categorical data were expressed as percentage. Antibody titer was reported as geometric mean. Positive rate of autoantibody was described as the frequency of autoantibody. For two-group comparison, we used the Student’s t test or Mann-Whitney U test for continuous variables and the chi-square test or Fisher’s exact test for categorical variables. For three-group comparison, Kruskal-Wallis test was used. Correlation between the levels of serum anti-β_1_-AABs or anti-M_2_R-AABs and the echocardiographic and clinical indices were assessed by Spearman’s correlation. Risk factor analysis was performed by univariate and multivariate logistic analysis. All tests were 2-tailed, and statistically significant difference was considered at *P* < 0.05. Statistical analysis was performed using SPSS Base 16.0 statistical software package (SPSS, Inc., Chicago, Illinois).
